# The Fabrication and Characterization of Self-Powered P-I-N Perovskite Photodetectors Using Yttrium-Doped Cuprous Thiocyanate

**DOI:** 10.3390/mi16060666

**Published:** 2025-05-31

**Authors:** Jai-Hao Wang, Bo-Chun Chen, Sheng-Yuan Chu

**Affiliations:** 1 Department of Electrical Engineering, National Cheng Kung University, Tainan 701, Taiwanilove61138@gmail.com (B.-C.C.); 2 Center for Micro/Nano Science and Technology, National Cheng Kung University, Tainan 701, Taiwan

**Keywords:** zinc oxide, self-powered photodetector, cuprous thiocyanate, yttrium

## Abstract

In the first part of this study, Y_2_O_3_-doped copper thiocyanate (CuSCN) with different x wt% (named CuSCN-xY, x = 0, 1, 2, and 3) films were synthesized onto ITO substrates using the spin coating method. UV-vis, SEM, AFM, EDS, and cyclic voltammetry were used to investigate the material properties of the proposed films. The conductivity and carrier mobility of the films increased with additional yttrium doping. It was found that the films with 2% Y_2_O_3_ (CuSCN-2Y) have the smallest valence band edges (5.28 eV). Meanwhile, CuSCN-2Y films demonstrated the densest surface morphology and the smallest surface roughness (22.8 nm), along with the highest conductivity value of 764 S cm^−1^. Then, P-I-N self-powered UV photodetectors (PDs) were fabricated using the ITO substrate/ZnO seed layer/ZnO nanorod/CsPbBr_3_/CuSCN-xY/Ag structure, and the characteristics of the devices were measured. In terms of response time, the rise time and fall time were reduced from 26 ms/22 ms to 9 ms/5 ms; the responsivity was increased from 243 mA/W to 534 mA/W, and the on/off ratio was increased to 2.47 × 10^6^. The results showed that Y_2_O_3_ doping also helped improve the P-I-N photodetector’s device performance, and the mechanisms were investigated. Compared with other published P-I-N self-powered photodetectors, our proposed devices show a fairly high on/off ratio, quick response times, and high responsivity and detectivity.

## 1. Introduction

With the evolution of human society and technological advances, sensors with low response times and high on/off ratios are becoming increasingly important. Self-powered photodetectors that do not require additional energy have also attracted widespread attention [1,2]. Photodetectors can be divided into infrared, visible light, and ultraviolet light sensors based on their wavelength bands. Ultraviolet light sensors have a wide range of applications, ranging from military missile tracking, communication, and flame detectors to ozone layer rupture detectors and ultraviolet sensing. Reported self-powered UV photodetectors have largely comprised P-N or P-I-N structures [3,4,5,6,7,8].

In P-I-N self-powered UV sensors, most of the materials used for the N layer include Ga_2_O_3_ [9], TiO_2_ [10], and ZnO [11,12]. Meanwhile, the materials used in the P layer could be MoO_3_ [13], NiO [14], CuO [15], or organic materials [16] such as Spiro-OMeTAD [17]. Lastly, the materials used in the I layer could be PEDOT [18], CsPbCl_3_ [19], and CsPbBr_3_ [20].

Among N-type materials, zinc oxide (ZnO) offers high stability, low cost, and a large bandwidth of 3.37 eV; furthermore, it belongs to the hexagonal close-packed (HCP) structure in the hexagonal crystalline system [21,22]. In addition, depending on the doping material, different optical bandgap energies of the zinc oxide can be obtained, and the nanostructures can be adjusted. Zinc oxide also has other advantages, such as high carrier mobility, low preparation temperature, non-toxicity, and ease of preparation, among others [23].

In layer I, the organic and inorganic perovskite materials [24,25] should have a high light absorption capacity, generate more photo-induced carriers, improve the separation efficiency of the carriers, and reduce the recombination. To date, there have been various methods employed to deposit the perovskite layer, including the sol–gel method, solid-state reaction, wet impregnation method, and spray drying method [26]. However, organic perovskite materials may degrade the composition and structure, as well as cause thermal and chemical instability due to moisture or oxygen in the manufacturing process. Therefore, the emergence of fully inorganic calcite materials, such as CsPbI_3_ and CsPbBr_3,_ significantly improves the thermal and optical stability of illumination [27,28,29].

Fanglin Wang et al. proposed an asymmetric Y_2_O_3_-coated BFBD device in which the channel is covered by a layer of an Y_2_O_3_ film and an overlap between the Sc electrode and the Y_2_O_3_ film is designed. The Y_2_O_3_ film provides p-type doping to the channel. The overlap section increases the length of the base of the pn junction, and the diffusion current of holes is suppressed. Doping from the Y_2_O_3_ film provides a large forward current and stable photocurrent generation [30].

For the P-type layer, the most typical material for the hole transport layer is Spiro-OMeTAD. However, it is expensive and unstable. On the other hand, CuSCN, which is not expensive, has become one of the most desirable candidates to replace Spiro-OMeTAD. Copper thiocyanate (CuSCN) is an inorganic p-type semiconductor with the properties of high optical transparency, a wide bandgap (>3.4 eV), and good thermal stability [31,32], which makes CuSCN an ideal hole transport layer (HTL) for applications such as perovskite solar cells, organic photovoltaic devices, and organic light-emitting diodes [33,34,35]. The deposition of CuSCN thin films mainly includes electrochemical deposition, spray-coating, spin-coating, drop-casting, and ink-jet printing. However, the mobility of CuSCN films still needs to be improved. Doping with Co, Zn, F4TCNQ, and Cl_2_ can improve the conductivity of CuSCN [35,36,37]. On the other hand, Y_2_O_3_ is a transparent oxidized material with good thermal stability, which can be used as a surface passivation layer to improve surface defects and enhance the conductivity of the material [38,39]. However, the Y_2_O_3_ doping effects of CuSCN films are seldom discussed, which is one of the motivations of this study.

In this experiment, we continue our previous work [40]. Firstly, we prepared CuSCN doped with different proportions of Y_2_O_3_ via the sol–gel method to improve the hole mobility; then, we fabricated an ITO substrate/ZnO seed layer/ZnO nanorod/CsPbBr_3_/CuSCN-xY/Ag structure using the proposed CuSCN-based films as a P-type hole transport layer, which effectively improved the response of the device. The response time, switching ratio, and responsiveness of the device were effectively improved. The related mechanisms were also analyzed in detail.

## 2. Experimental Method and Characterization

Part 1: CuSCN film synthesis

We pretreated the ITO glass substrate using a series of organic solvents (including deionized water, ethanol, and isopropanol) for the ITO section. We treated the ITO surface with UV–ozone for 20 min.

**Figure 1:**
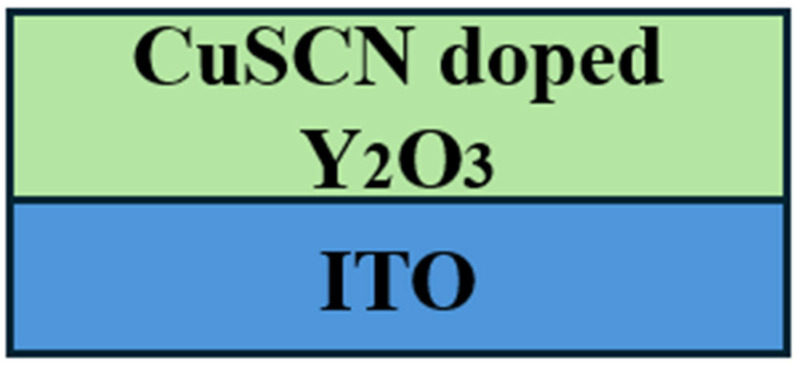
Schematic diagram of thin films of CuSCN doped with Y_2_O_3_.

Figure 1 shows the schematic diagram of CuSCN films doped with Y_2_O_3_. CuSCN films were synthesized using the spin-coating method. CuSCN powders of 140 mg were dissolved in 4 mL of diethyl sulfide with 0% wt, 1% wt, 2% wt, and 3% wt of Y_2_O_3_ before being spin-coated on the ITO substrates for 30 s at 5000 rpm. Then, the samples with a thickness of 100 nm were heated to 80 °C for 30 min.

Part 2: P-I-N photodetector fabrications

**Figure 2:**
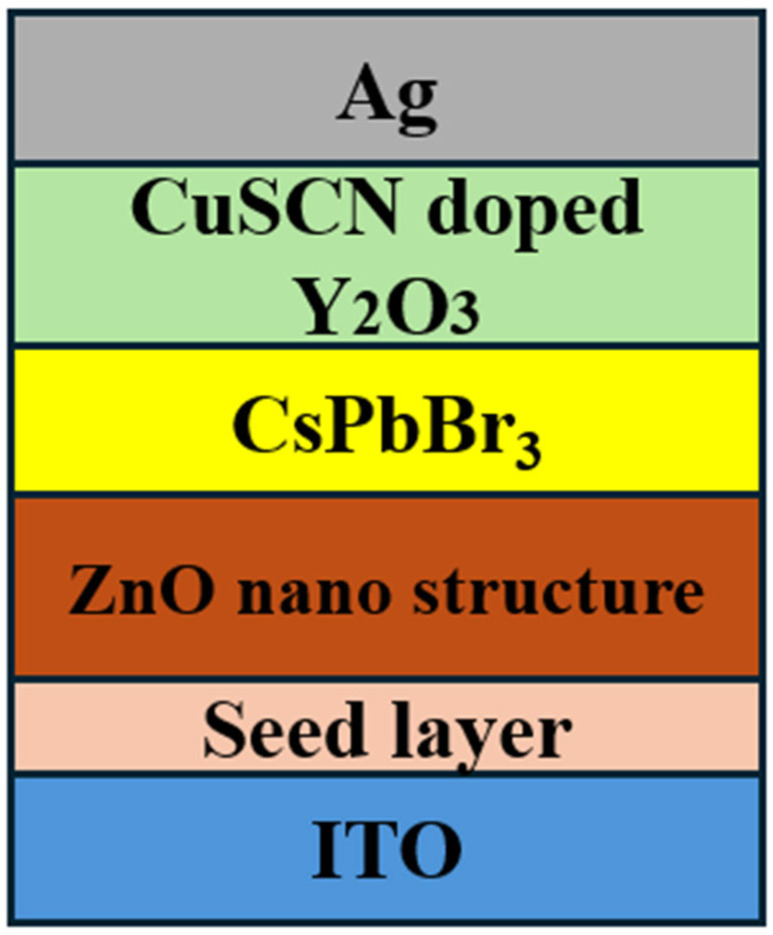
Schematic diagram of device structure.

Figure 2 shows the structure of the device. The zinc oxide seed layer was prepared using the atomic layer deposition method (ALD). Diethylzinc (DEZn) and water vapor (DI) were used as Zn and O precursors, respectively. Argon gas was injected into the ALD chamber for purging after each reaction step, and the pulse duration was set to 0.1 s for DEZn and 5 s for DI. The total number of cycles for the growth of the ZnO seed layer was maintained at approximately 500 per growth in order to achieve a thickness of around 100 nm. The temperature of the substrate was set at 80 °C.

In the ZnO nanostructure section, the hydrothermal method was used, with 1.09 g of zinc acetate and 0.41 g of hexamethylamine added to 100 mL of deionized water. The samples with the ZnO seed layer were placed into a hydrothermal autoclave reactor. Then, it was heated in an oven at 80 °C for 4 h.

For the perovskite film section, a PbBr_2_ solution consisting of 3.67 g PbBr_2_ was poured into a bottle, along with 4 mL of DMF and 6 mL of DMSO mixed solvent. The temperature was set to 75 °C. The CsBr solution consisted of 1.48 g of CsBr poured into a 100 mL volumetric flask, with anhydrous methanol added before the flask was closed tightly. The sample was spin-coated with approximately 100 μL of PbBr_2_ at 3000 rpm for 30 s and annealed for 30 min at 75 °C. Then, the sample was soaked in CsBr for 15 min and annealed for 5 min at 250 °C. The thickness of the film was about 400 nm.

CuSCN films were synthesized via the spin-coating method; CuSCN powder was dissolved in diethyl sulfide and mixed with different proportions of yttrium in spin-coats at 5000 rpm for 30 s. Finally, the 100 nm silver electrode was deposited at a plating rate of 4.0 Å/s [1].

Field emission scanning electron microscopy (SEM, HITACHI SU8000, The HITACHI SU8000 is manufactured in Japan by HITACHI.) was used to characterize the morphology of the CuSCN-xY films. A UV-Vis spectrophotometer (Hitachi U-3000) was used to measure the absorption spectra of the CuSCN-xY films at different doping concentrations. The elemental composition of CuSCN-xY films was analyzed using a multi-function environmental field emission scanning electron microscope (EDS, HITACHI SU-5000). The roughness of the CuSCN-xY films was measured using an atomic force microscope (AFM). Conductivity was measured using 4155C and determined via the two-point probe method. The electrochemical properties of the samples were investigated via cyclic voltammetry (CV) tests using a software-controlled conventional three-electrode electrochemical cell (Autolab PGSTAT302N) consisting of the as-prepared samples as the working electrode, Ag/AgCl as the reference electrode, a Pt foil as the counter electrode, and a 0.1 M KCl solution as the electrolyte at room temperature. A potential window in the range from 0 V to +0.4 V was used in all the measurements. The CV measurements were performed at different scan rates at 100 mV/s. The current/time (I-t) characteristics were measured using a Hitachi F-7000 as the light source and a 4155C as the current meter. The monochromatic light provided by the spectrometer was used to measure the response time, photocurrent, and dark current of the PD. The PD was irradiated with low-intensity UV light (2.558 mW/cm^2^).

## 3. Discussion and Results

**Figure 3:**
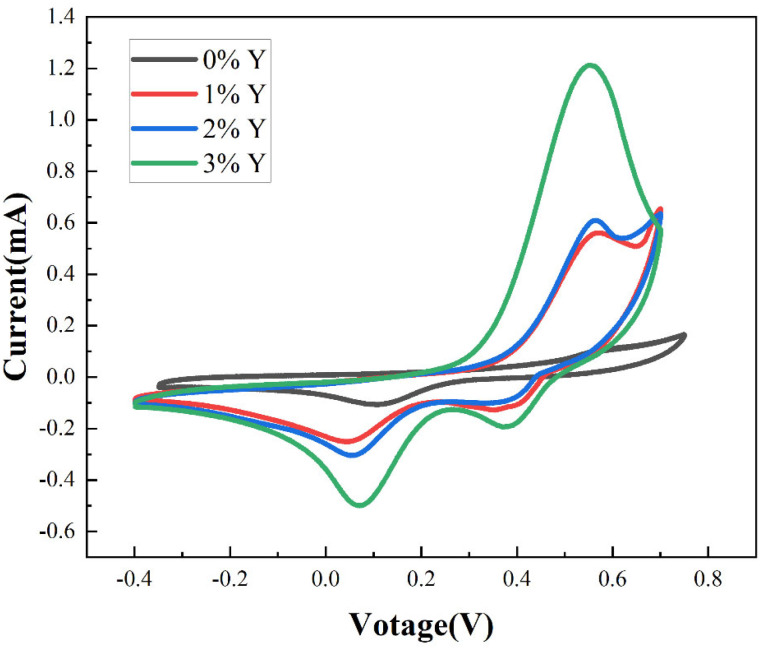
Cyclic voltammetry plots of the films.

Figure 3 shows the cyclic voltammetry diagram of the proposed CuSCN-based films. The increase in current with increasing doping concentration indicates an enhancement of redox reactions, which is presumed to result from Y doping increasing the hole density in the CuSCN electrode. From Figure 3, the oxidation onset value E_ox_ of the films can be obtained as shown in Table 1.

The valence band edges of the films were calculated according to Equation (1) as follows:

Valence band edge = −{(0.22 + 4.5) + E_ox_} (1)

where 4.5 is the difference between the potential of the standard hydrogen electrode and vacuum, and 0.22 is the potential difference between silver chloride and the standard hydrogen electrode. Table 1 shows the calculated valence band edge of the proposed films. We can see from Table 1 that the oxidation onset value tends to decrease as the doping concentration is increased, and the valence band edge decreases from 5.32 eV (CuSCN-0Y) to 5.26 eV (CuSCN-3Y). Yttrium exhibits multi-coordination, in contrast to the single coordination observed in copper within CuSCN. The unique properties of yttrium facilitate robust bonding with thiocyanate counter ions, consequently leading to a weakened SCN–Cu bond. This phenomenon introduces alternative energy pathways for electronic excitation, resulting in reduced photon energy required for excitation within a diminished modified band gap [41,42].

**Figure 4:**
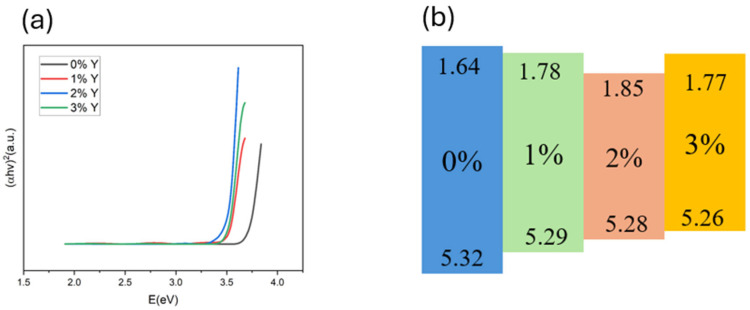
(**a**) Tauc plots and (**b**) energy band plots of CuSCN-based films with different Y_2_O_3_ doping concentrations.

Figure 4a shows the energy bandgaps derived from the absorption spectra of CuSCN films. The Tauc method is used to obtain the bandgap. We observe that the energy gap of CuSCN-0Y is 3.68 eV; with the doping of Y_2_O_3_, the minimum energy gap of 3.43 eV is obtained at 2%, which indicates that the doped of Y_2_O_3_ decreases the band gap of CuSCN. As the doping concentration increases, the valence band also decreases and reduces the energy gap, as shown in Table 1. The conductivity at 2% doping Y_2_O_3_ measured using 4155C shows a maximum value of 764 S cm^−1^. Additionally, we obtained the band gap using the Tauc equation and determined the conduction band based on the previously obtained valence band. Using the band gap, valence band, and conduction band, we constructed the diagram shown in Figure 4b.

**Figure 5:**
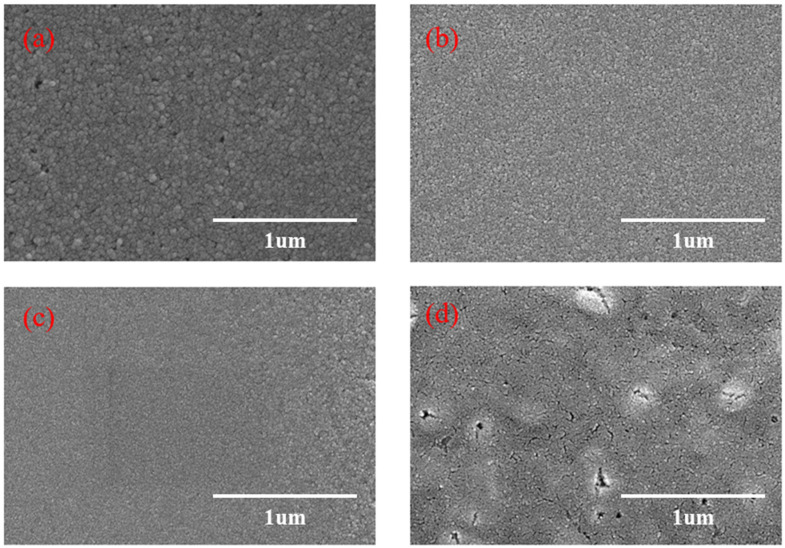
SEM images of CuSCN films with different Y_2_O_3_ dopings: (**a**) 0% Y_2_O_3,_ (**b**) 1% Y_2_O_3,_ (**c**) 2% Y_2_O_3,_ and (**d**) 3% Y_2_O_3_.

The SEM photographs of CuSCN films are shown in Figure 5a–d, and it can be found that the quality of the films becomes better when the doping ratio increases; the highest quality of the films is obtained when the doping is 2% Y_2_O_3_. When doped with 1% Y_2_O_3_, the surface morphology is improved. When doped with 2% Y_2_O_3,_ a denser film is formed on the surface. When doped with 3% Y_2_O_3_, the grain size and distribution are differentiated, and more clusters are formed on the surface.

Table 2 shows the EDS of CuSCN films. As the Y_2_O_3_ doping ratio increases, it can be seen that the atomic percentages of mixed sulfur atoms, carbon atoms, and nitrogen atoms are basically the same for each of them, whereas the atomic percentage of Cu decreases from 19.61 at.% to 9.74 at.%, 9.25 at.%, and 8.21 at.% when Y_2_O_3_ content is changed from 0 at.%, 1.01 at.%, 2.10 at.%, and 2.83 at.%, indicating that Y atoms replace some of the Cu atoms, resulting in a decrease in the Cu percentage.

**Figure 6:**
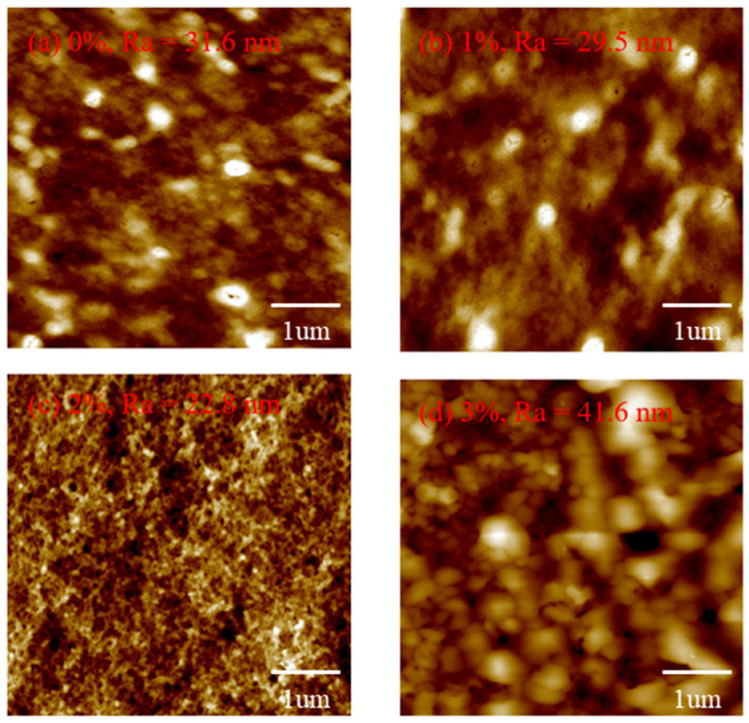
AFM diagrams of (**a**) 0%, (**b**) 2%, (**c**) 2%, and (**d**) 3% doping ratios of Y_2_O_3_.

**Figure 7:**
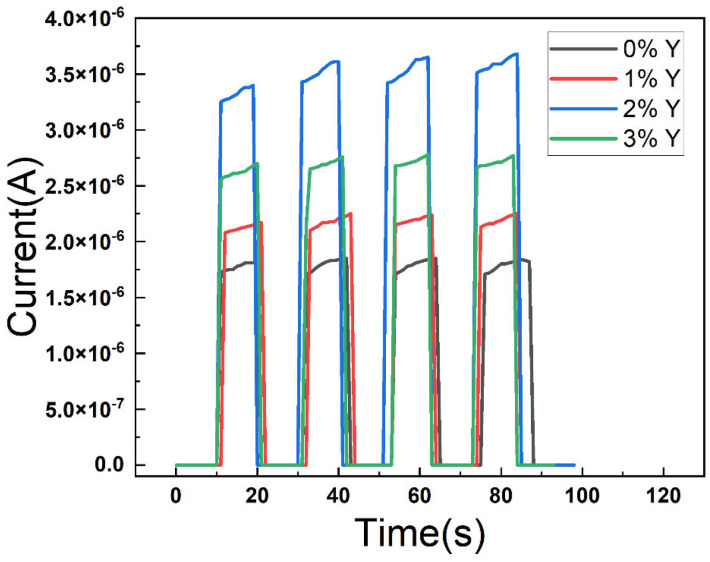
I-t diagram of the devices at 0 V bias voltage.

Figure 6 shows the AFM diagram of all the films. It is observed that the R_a_ of CuSCN-0Y and CuSCN-2Y is 31.6 and 22.8 nm. When we doped 3% Y_2_O_3_, the roughness increased to 41.6 nm, which is in agreement with the SEM data. From the SEM and AFM results, it is suggested that the maximum solubility of the Y_2_O_3_ doping is about 2 wt%. Figure 7 shows the I-t diagram of the devices with different CuSCN HTLs measured with a light source at 360 nm. It can be observed that the photocurrent is 1.26 × 10^−6^ A for the device using CuSCN-0Y as HTL, and the highest photocurrent of 3.51 × 10^−6^ A is obtained when CuSCN-2Y is used. Detailed device performances are shown in Table 3. The highest on/off ratios of 2.47 × 10^6^ were obtained at 2% Y_2_O_3_. After 3% doping of Y_2_O_3_, the on/off ratio decreased, which may be due to defects in the surface clusters. This corresponds to the poor surface roughness at 3%, which affects the interfacial quality between the HTL and the active layer. An excessively rough surface may lead to an increase in interfacial defects, which, in turn, leads to an increase in the carrier recombination rate and a decrease in the photogenerated charge collection efficiency. In order to further evaluate the detection performance of each device in a comprehensive manner, the responsivity can be calculated by the equation R (λ) = (I_λ_ − I_d_)/(P_λ_ S), where R (λ) is the responsivity, I_λ_ is the UV-irradiated photocurrent, I_d_ is the dark current, and P_λ_ S represents the illumination power intensity and effective light area. The other important PD parameter, detectivity, is based on the equation D = (R × S^1/2^)/(2 eI_d_)^1/2^, where e is the Planck constant. We obtained a detectivity of 2.03 × 10^12^ Jones in CuSCN-2Y, while responsivity was 685 mA/W at 0 V bias, and the enhancement of these characteristics is attributed to increased mobility resulting in faster carrier movement.

**Figure 8:**
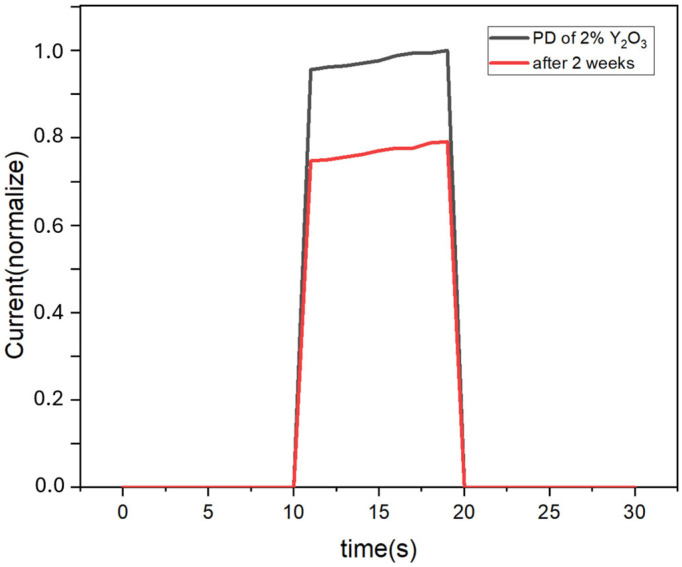
I-t spectra of the devices using CuSCN-2Y as the HTL after 2 weeks in the atmospheric environment.

Figure 8 demonstrates the I-t spectra of the devices after 2 weeks in the atmospheric environment. When the devices are placed for two weeks, the current value becomes about 0.8 times the original one. This is because CuSCN is a hydrophobic material that protects the perovskite layer from water vapor erosion.

Table 4 shows the device performance comparison with the published P-I-N self-powered UV photodetectors. Our devices show the highest on/off ratio, quickest rise time/fall time, and highest responsivity and detectivity. The best rise time/fall time we obtained was 9 ms/5 ms. Responsivity was 685 mA/W, and we obtained a detectivity of 2.03 × 10^12^ Jones.

## 4. Conclusions

In the first part of this study, copper thiocyanate (CuSCN) doped with Y_2_O_3_ was synthesized using the spin coating method, and the EDS analysis proved that Y_2_O_3_ was doped into CuSCN to increase the electrical conductivity by replacing copper. We determined that the Y_2_O_3_ mixed at 2% has the smallest valence band edge (5.28 eV). Meanwhile, with the increase in the mixing ratio, the densest surface morphology and the most compact surface morphology were obtained at 2%. In terms of conductivity, the highest value of 764 S cm^−1^ was obtained.

Then, we fabricated a P-I-N self-powered UV photodetector using CuSCN-based films as the HTL. The rise and fall times were reduced to 9 ms/5 ms in response time; the responsivity increased to 685 mA/W; and the on/off ratio increased to 2.47 × 10^6^. These results show that mixing Y_2_O_3_ enhances the device performance of P-I-N photodetectors by improving the carrier mobility of the HTL.

## Figures and Tables

**Table 1:** Energy bandgaps, valence/conduction band edges, and conductivity of CuCSN-based films.

Doped Ratio	E_ox_	Eg (eV)	Valence Band Edge (eV)	Conduction Band Edge (eV)	Conductivity (S cm^−1^)
0%	0.60	3.68	5.32	1.64	218
1%	0.57	3.51	5.29	1.78	516
2%	0.56	3.43	5.28	1.85	764
3%	0.54	3.49	5.26	1.77	654

**Table 2:** EDS of CuSCN-based films.

Doping Ratio	Cu (at.%)	S (at.%)	C (at.%)	N (at.%)	Y_2_O_3_ (at.%)
0%	19.61	14.9	41.06	23.73	0
1%	9.74	6.23	38.42	44.6	1.01
2%	9.25	6.1	38.33	44.21	2.10
3%	8.21	5.96	38.3	44.7	2.83

**Table 3:** Parameters of device performances with different CuSCN-based HTLs.

Y_2_O_3_	Light Current (μA)	Dark Current (pA)	On/Off Ratio (10^6^)	Rise Time (ms)/Fall Time (ms)	Responsivity (mA/W)	Detectivity (10^12^ Jones)
0%	1.26	1.36	0.926	26/22	452	1.37
1%	2.17	1.57	1.38×	23/19	521	1.47
2%	3.51	1.42	2.47×	9/5	685	2.03
3%	2.62	1.52	1.72×	20/17	586	1.68

**Table 4:** Comparison with other published P-I-N self-powered photodetectors.

	On/Off Ratio	Responsivity (mA/W)	Rise Time /Fall Time	Detectivity (Jones)
ITO/ZnO/CsPbBr_3_/CuSCN mixing Y_2_O_3_/Ag [This work] (0v@360 nm)	2.47 × 10^6^	685	9 ms /5 ms	2.03 × 10^12^
p-GaN/i-Ga_2_O_3_/n-Ga_2_O_3_ (0v@360 nm) [43]	1.88 × 10^4^	49	7 ms /19 ms	1.22 × 10^12^
Graphene/γ-Ga_2_O_3_/SiC (0v@250 nm) [44]	32	5.8	30 ms /196 ms	7.6 × 10^10^
Graphene/MgZnO/SiC (0v@260 nm) [45]	226.4	2.64	78.2 ms /162 ms	-
ITO/ZnO/CsPbBr_3_/CuSCN/Ag [This work] (0v@360 nm) [40]	3.96 × 10^6^	497.9	<10 ms	6.81 × 10^10^
ZnO NRs/CsPbBr_3_/MoO_3_/Au [46]	450	300	200 ms/500 ms	-

## Data Availability

The data presented in this study are available on request from the corresponding author due to privacy.
